# Changes during Food Freezing and Frozen Storage

**DOI:** 10.3390/foods10112525

**Published:** 2021-10-21

**Authors:** Maria Giannakourou, Petros Taoukis

**Affiliations:** 1 Department of Food Science and Technology, University of West Attica, 12243 Egaleo, Greece; 2 Laboratory of Food Chemistry and Technology, School of Chemical Engineering, National Technical University of Athens, 15780 Athens, Greece; taoukis@chemeng.ntua.gr

Multiple factors can directly influence frozen food quality, during processing and post-processing, in the cold chain. There is a great interest in investigating, understanding, and ultimately quantitatively expressing the effect of the quality determining factors with regards freezing method applied, ice crystal morphology, food microstructure, glass transition phenomena, temperature and temperature fluctuations during distribution and storage. This can contribute to the improvement of the freezing process and the subsequent handling, aiming at optimum frozen product quality from process to final use or consumption. In this context, this Special Issue “Changes during Food Freezing and Frozen Storage” comprises research papers and review articles focused on the different phenomena observed during food freezing and subsequent storage.

Roos (2021) [1] authoritatively and comprehensively covered glass transition and re-crystallization phenomena of frozen materials and their effect on frozen food quality. The article highlights factors leading to the formation of non-crystalline, freeze-concentrated structures in food freezing and the physicochemical properties of frozen materials with non-crystalline phases, including solute and solvent recrystallization phenomena affecting frozen food characteristics and quality.

Neri et al. (2020) [2] critically reviewed the effect of cryoprotectants, freezing process and frozen storage on the antioxidant activity of plant foods. Cryoprotectants, and their role in the preservation of the antioxidant activity of vegetables and fruits and their main derivatives (e.g., juices or pulps), is presented in depth.

Giannakourou et al. (2020) [3] reviewed research progress on the osmo-dehydrofreezing of food products, aiming to improve frozen food quality and stability, during long-term frozen storage. The effect of this combined process on different quality indices was approached in an integrated approach, aiming at an efficient design of the process and assessment of the osmo-dehydrofrozen product quality during processing, storage, and distribution in the cold chain.

By implementation of kinetics on quality degradation and shelf life prediction of frozen foods, Tsironi et al. (2021) [4] developed mathematical models to describe the effect of variable storage conditions on the quality of frozen fish and validated their applicability as effective predictive tools for frozen chain monitoring and management.

The subject of shelf life estimation of frozen meat products was studied by Leygonie et al. (2020) [5]. The interaction between different rates of freezing and thawing on whole ostrich moon steaks was investigated, in order to establish a combination or singular main effect that minimizes thaw loss and maximizes the retail display shelf life regarding moisture loss, color, lipid oxidation, and tenderness.

The reliable prediction of frozen food shelf life, at any point in the actual cold chain, was addressed by Giannakourou and Taoukis (2020) [6]. A holistic approach to shelf life calculations through a double Monte Carlo technique was applied to frozen food kinetics data, accounting for kinetic parameter uncertainty and temperature variability at all stages of the cold chain. Assessment of the factors affecting shelf life calculations through a sensitivity analysis provides a tool for reliable management and optimization of the frozen food cold chain.

In summary, the Special Issue “Changes during Food Freezing and Frozen Storage” evidences the great importance of investigating the impact of the freezing process and frozen storage conditions on food quality and shelf life and building this knowledge into effective mathematical tools that can lead into a quantitative assessment of process and post-process quality determining parameters. This will ultimately support the optimized management of the cold chain from production to use.
